# IRS-2 rs1805097 polymorphism is associated with the decreased risk of colorectal cancer

**DOI:** 10.18632/oncotarget.15342

**Published:** 2017-02-15

**Authors:** Jiefeng Yin, Zhe Zhang, Huajun Zheng, Lei Xu

**Affiliations:** ^1^ General Surgery Department, Tongde Hospital of Zhejiang Province, Hangzhou, Zhejiang, China; ^2^ Department of Digestion, The Second Affiliated Hospital of Zhejiang Chinese Medical University, Hangzhou, Zhejiang, China

**Keywords:** IRS-2, single nucleotide polymorphism, colorectal cancer, meta-analysis, rs1805097

## Abstract

Recent studies explored the association between insulin receptor substrate-2 (IRS-2) gene rs1805097 polymorphism and colorectal cancer (CRC) with contradictory findings. Therefore, we conducted a comprehensive meta-analysis by searching the databases of PubMed and Embase. Pooled odds ratios (ORs) and 95% confidence intervals (CIs) were calculated by using fixed-effect or random-effect models. A total of 5 citations containing 6 case-control studies involving 4,333 cases and 5,333 controls were included. Our data indicated that IRS-2 rs1805097 polymorphism was associated with decreased risk of CRC. Stratification analysis of ethnicity found that rs1805097 polymorphism decreased the risk of CRC among Americans. Stratification analysis of cancer type suggested that this polymorphism decreased the risk of colon cancer. In summary, this meta-analysis indicates that IRS-2 gene rs1805097 polymorphism plays an important role in the pathogenesis of CRC.

## INTRODUCTION

Colorectal cancer (CRC) is the second most commonly diagnosed cancer worldwide [1]. CRC is one of the primary causes of cancer-related mortality. To date, the etiology of CRC is still unclear. Some environmental factors including diet, cigarette smoking, physical inactivity, and alcohol consumption, are considered to influence the risk of CRC [2]. Studies demonstrated that those environmental factors through the insulin pathway are significantly associated with the risk of CRC [3, 4]. Several researches provided evidence to support that insulin is associated with the risk of CRC [3, 4]. Some researchers reported that hyperinsulinaemia and type 2 diabetes influence the risk of colon cancer [5]. Animal research found that insulin enhances the growth of aberrant crypt foci, CRC precursor lesions, and increases the number and the size of the tumors [4].

Insulin receptor substrates (IRSs) are involved in insulin signaling pathway [6]. IRS-2 plays an important role in glucose metabolism, tumor progression, and metastasis [7]. A host of studies [8–12] investigated the association between IRS-2 gene rs1805097 polymorphism and CRC risk, but with conflicting findings. These conflicting and inconclusive results may due to clinical heterogeneity, diverse ethnic populations, different tumor types, and small sample sizes. Therefore, we performed a comprehensive meta-analysis to clarify the possible association between IRS-2 gene rs1805097 polymorphism and CRC risk.

## RESULTS

### Characteristics of the included studies

**Figure 1:**
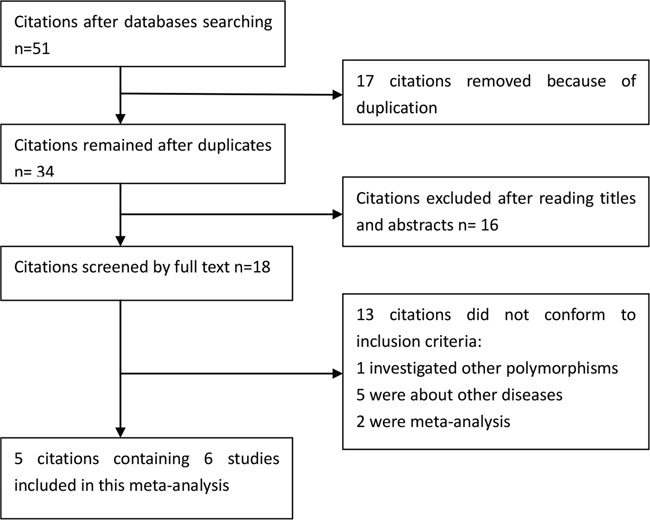
Selection for eligible publications included in this meta-analysis

We yielded a total of 51 citations after initial search. 33 citations were removed after removing duplicates and screening the titles and abstracts. 18 citations were selected for further full text review. 13 citations were excluded: 1 investigated other polymorphisms; 5 were about other diseases; 2 were meta-analyses. Finally, 5 citations [8–12] containing 6 studies (4,333 cases and 5,333 controls) were included in this meta-analysis. Selection for eligible studies included in this meta-analysis was presented in Figure 1. The characteristics of included studies are summarized in Table 1. The Newcastle-Ottawa Scale (NOS) scores of all included studies ranged from 5 to 8 stars. All studies conformed to the Hardy–Weinberg equilibrium (HWE).

**Table 1:** Characteristics of included studies

Author and year	Country	SOC	Genotype methods	Ethnicity	Case			Control			HWE	NOS
					GG	GA	AA	GG	GA	AA		
Mahmoundi_2014	Iran	HB	PCR	Caucasian	109	118	34	139	153	47	Y	6
Yukselogu_2014	Turkey	HB	PCR-RFLP	Caucasian	79	58	24	88	85	24	Y	7
Pechivanis_2007	Czech	HB	PCR	Caucasian	211	277	81	268	309	106	Y	7
Samowitz_2006	USA	HB	PCR	American	718	657	197	829	906	229	Y	8
Slattery_2004a	USA	PB	PCR	American	467	409	128	481	552	134	Y	6
Slattery_2004b	USA	PB	PCR	American	325	343	562	421	423	139	Y	6

Abbreviations: SOC, source of control; PB, population-based controls; HB, hospital-based controls; HWE, Hardy–Weinberg equilibrium; NOS, Newcastle-Ottawa Scale.

### Meta-analysis of IRS-2 gene rs1805097 polymorphism

**Figure 2:**
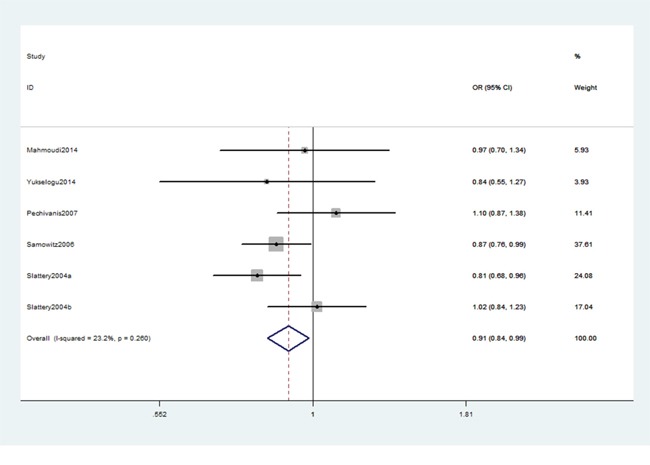
Forest plot shows odds ratio for the associations between rs1805097 polymorphism and CRC risk (AA+GA vs. GG)

**Figure 3:**
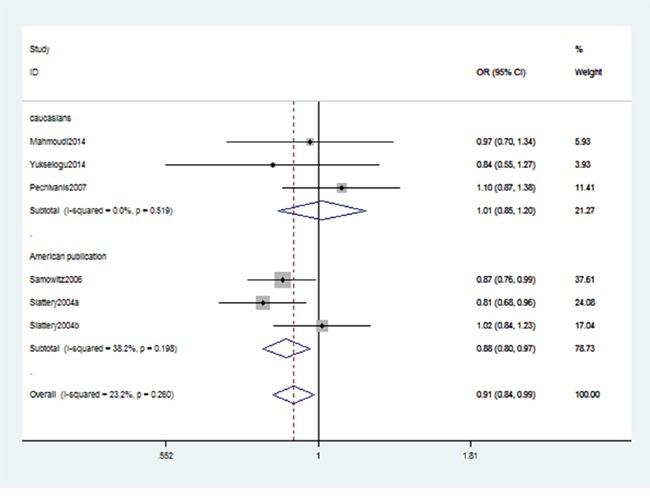
Stratification analyses by ethnicity shows odds ratio for the associations between rs1805097 polymorphism and CRC risk (AA+GA vs. GG)

**Figure 4:**
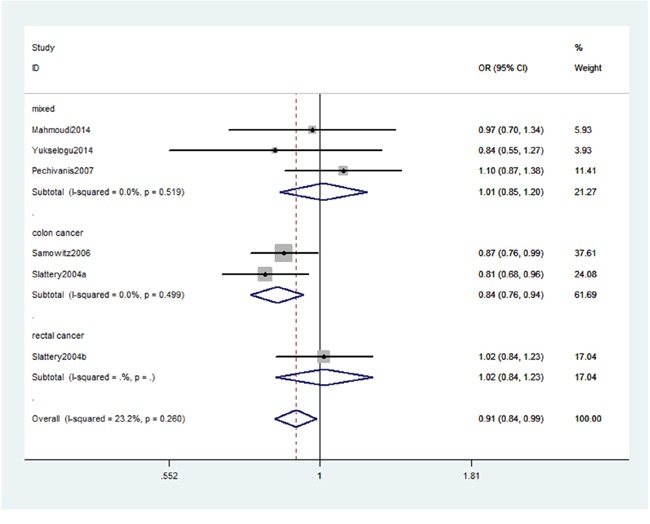
Stratification analyses of cancer type between rs1805097 polymorphism and CRC risk (AA+GA vs. GG)

As shown in Table 2, we detected an association between IRS-2 gene rs1805097 polymorphism and CRC risk (AA+GA vs. GG: OR, 0.91; 95% CI, 0.84–0.99, *P* = 0.022, Figure 2). Stratification analysis by ethnicity indicated that rs1805097 polymorphism was significantly associated with a decreased risk of CRC among Americans (AA+GA vs. GG: OR, 0.88; 95% CI, 0.80–0.97, *P* = 0.007, Figure 3), but not among other Caucasians. Stratification analysis of cancer type suggested that this polymorphism decreased the risk of colon cancer (AA+GA vs. GG: OR, 0.84; 95% CI, 0.76–0.94, *P* = 0.002, Figure 4). Regarding stratification analysis by source of control (SOC), no positive result was obtained in both population-based population and hospital-based population (Table 3).

**Table 2:** Meta-analysis of association between IRS-2 rs1805097 polymorphism and colorectal cancer risk

Comparison	OR(95%CI)	*P*-value	*P* for heterogeneity	I^2^ (%)	Model
A vs. G	0.96(0.90,1.01)	0.132	0.939	0	Fixed
AA+GA vs. GG	**0.91(0.84,0.99)**	0.022	0.260	23.2	Fixed
AA vs. GG+GA	1.02(0.91,1.15)	0.721	0.695	0	Fixed
AA vs. GG	0.97(0.86,1.10)	0.669	0.994	0	Fixed
GA vs. GG	0.91(0.79,1.05)	0.189	0.055	53.7	Random

**Table 3:** Summary of the subgroup analyses in this meta-analysis

Comparison	Category	Category	Studies	OR (95% CI)	*P*-value	*P* for heterogeneity
A vs. G	Ethnicity	Caucasians	3	1.00(0.88,1.13)	0.943	0.913
		Americans	3	0.94(0.88,1.01)	0.093	0.939
	Cancer type	mixed	3	1.00(0.88,1.13)	0.943	0.913
		Colon cancer	2	0.93(0.86,1.01)	0.078	0.696
		Rectal cancer	1	0.98(0.85,1.13)	0.772	<0.001
	SOC	HB	4	0.96(0.89-1.04)	0.350	0.892
		PB	2	0.94(0.86-1.04)	0.218	0.472
AA+GA vs. GG	Ethnicity	Caucasians	3	1.01(0.85,1.20)	0.884	0.519
		Americans	3	**0.88(0.80,0.97)**	0.007	0.198
	Cancer type	mixed	3	1.01(0.85,1.20)	0.884	0.519
		Colon cancer	2	**0.84(0.76,0.94)**	0.002	0.499
		Rectal cancer	1	1.02(0.84,1.23)	0.867	<0.001
	SOC	HB	4	0.92(0.83,1.02)	0.125	0.358
		PB	2	0.89(0.79,1.01)	0.082	0.076
AA vs.GA+ GG	Ethnicity	Caucasians	3	0.96(0.75,1.22)	0.732	0.625
		Americans	3	1.04(0.91,1.20)	0.541	0.422
	Cancer type	mixed	3	0.96(0.75,1.22)	0.732	0.625
		Colon cancer	2	1.10(0.94,1.29)	0.238	0.825
		Rectal cancer	1	0.89(0.67,1.18)	0.414	<0.001
	SOC	HB	4	1.03(0.88,1.20)	0.704	0.674
		PB	2	1.01(0.84,1.22)	0.920	0.225
AA vs.GG	Ethnicity	Caucasians	3	0.98(0.76,1.27)	0.875	0.900
		Americans	3	0.97(0.84,1.12)	0.687	0.898
	Cancer type	mixed	3	0.98(0.76,1.27)	0.875	0.900
		Colon cancer	2	0.99(0.84,1.17)	0.904	0.957
		Rectal cancer	1	0.91(0.68,1.23)	0.549	<0.001
	SOC	HB	4	0.99(0.84,1.17)	0.882	0.975
		PB	2	0.95(0.78,1.16)	0.662	0.718
GA vs.GG	Ethnicity	Caucasians	3	1.01(0.82,1.24)	0.929	0.292
		Americans	3	0.91(0.79,1.05)	0.099	0.062
	Cancer type	mixed	3	1.01(0.82,1.24)	0.929	0.292
		Colon cancer	2	0.81(0.72,0.90)	<0.001	0.428
		Rectal cancer	1	1.05(0.86,1.29)	0.635	<0.001
	SOC	HB	4	0.93(0.78,1.11)	0.404	0.145
		PB	2	0.89(0.65,1.22)	0.475	0.021

Abbreviations: SOC, source of control; PB, population-based controls; HB, hospital-based controls.

**Figure 5:**
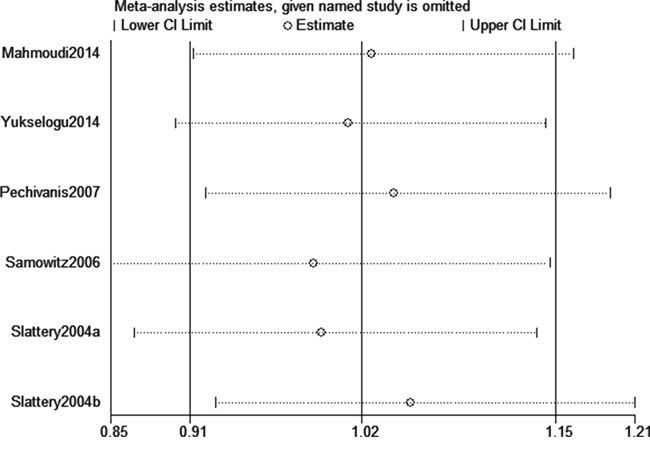
Sensitivity analysis about rs1805097 polymorphism and CRC risk (AA vs. GG+GA)

**Figure 6:**
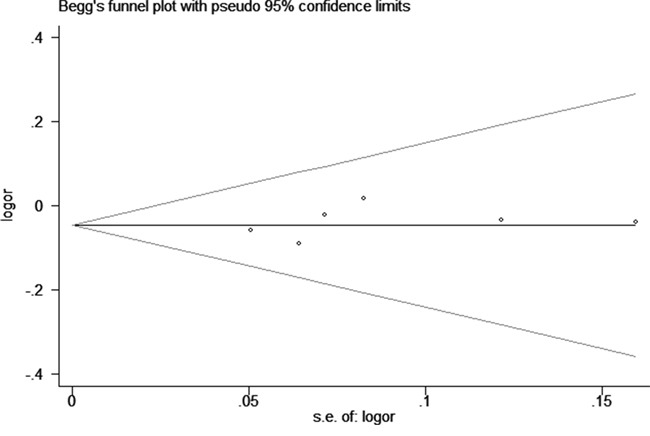
Begg's tests for rs1805097 polymorphism and CRC risk (A vs. G)

We assessed sensitivity by omitting each study once at a time in every genetic model for rs1805097 polymorphism. Our data indicated that the findings of this meta-analysis were stable and trustworthy (AA vs. GG+GA, Figure 5). Both Egger's and Begg's tests (A vs. G, Figure 6) revealed that there was no obvious publication bias for rs1805097 polymorphism (data not shown).

## DISCUSSION

In this current meta-analysis, we found that IRS-2 gene rs1805097 polymorphism decreased the risk of CRC. Stratification analysis revealed that rs1805097 polymorphism was associated with a decreased risk of CRC among Americans. In addition, stratification analysis of cancer type suggested that rs1805097 polymorphism decreased the risk of colon cancer.

Insulin, a hormone, controls the energy homeostasis by functioning on target tissues. Insulin increases cell proliferation and decreases apoptosis [4, 13]. Many studies indicated that hyperinsulinemia and insulin resistance (IR) are involved in the etiology of CRC [14]. Hyperinsulinemia interacting with obesity was an important risk factor for CRC [4, 14]. Larsson et al. found a relationship between diabetes and increased risk of CRC [13]. Insulin-like growth factor (IGF), insulin-like growth factor binding proteins (IGFBPs), insulin and IRS play crucial roles in the initiation of cell growth and CRC proliferation [15, 16]. IRS-2 mediates the major metabolic, proliferative, and antiapoptotic functions of the IGF1 [17, 18]. IRS-2 gene variants were reported to be involved in the modulation of IRS-1 or IRS-2 functions and could be relevant to colorectal tumorigenesis [19]. So far, a number of studies [8–12] investigated the association between IRS-2 gene rs1805097 polymorphism and CRC risk. However, these studies yield contradictory results. Thus, we conducted this current meta-analysis.

Previous meta-analysis by Hu et al. indicated that IRS-2 rs1805097 polymorphism was not associated with CRC risk [20]. They only included 4 studies. To date, two studies [9, 11] have been reported in recent years since the meta-analysis [20]. In analysis of all included studies, our meta-analysis found that rs1805097 polymorphism was associated with decreased CRC risk. We believed that Hu et al. falsely extracted the genotype numbers of cases and controls from an American study [8]. Actually, the genotype numbers of this study were as following: colon cancers (GG=467, GA=409, AA=128), controls (GG=481, GA=552, AA=134); rectal cancers (GG=325, GA=343, AA=98), controls (GG=421, GA=423, AA=139). In addition, the above false data explained the reason why the HWE value in this American study was wrong [8]. According to our data, the study [8] by Slattery et al. conformed to HWE. We also conducted stratification analysis of cancer type, which was not performed by Hu et al. [20]. Our data suggested that rs1805097 polymorphism was associated with a decreased risk of colon cancer.

We believe our meta-analysis more robust than previous meta-analysis by Hu et al. [20]. First, we included 2 extra studies and the sample size of this meta-analysis was larger than previous meta-analysis. Second, sensitivity analysis indicated that our data about rs1805097 polymorphism were trustworthy and stable. Third, the power analysis indicated that this meta-analysis had a power of 96.9% to detect the effect of rs1805097 polymorphism on CRC risk with an OR of 0.91.

Several potential limitations should be addressed in this meta-analysis. First, due to limited data, we could not investigate the association between CRC and other potential factors, such as age and sex. Second, our results were based on unadjusted estimates for confounding factors, which might have affected the final findings. Third, we could not assess potential gene-gene and gene-environment interactions because of the lack of relevant data. Fourth, the sample size of this meta-analysis is not very large, and the number of included studies is small. Fifth, the findings of the stratified analyses should be interpreted with caution because of limited sample sizes.

In conclusion, this meta-analysis indicates that IRS-2 gene rs1805097 polymorphism decreased the risk of CRC. Further studies are necessary to validate whether rs1805097 polymorphism contributes to CRC susceptibility in other ethnic groups.

## MATERIALS AND METHODS

### Literature search and inclusion criteria

We systematically searched the PubMed and Embase to identify studies through August 19, 2016. The following search terms were used: “cancer,” ‘‘carcinoma,’’ “neoplasm,’’ ‘‘tumor,’’ ‘‘Insulin Receptor Substrate 2,’’ ‘‘IRS-2,’’ and ‘‘IRS 2′’. No restrictions were placed on the literature search. Reference lists were identified by hand screening. The identified studies conformed to the following criteria: (1) studies that evaluated the association between IRS-2 gene rs1805097 polymorphism and CRC risk, (2) study provided sufficient data to calculate the odds ratios (ORs) and 95% confidence intervals (CIs), (3) case-control study.

### Data extraction and quality assessment

Relevant information was carefully extracted from all eligible studies. The extracted information from all eligible studies including: name of first author, publication year, country of origin, ethnicity, genotype methods and genotype numbers of cases and controls. Two authors independently performed the extraction of data and assessed the study quality based on the NOS [21]. All disagreements were discussed and resolved with consensus.

### Statistical analyses

All statistical analyses were performed using the Stata 11.0 software (StataCorp, College Station, TX, USA). ORs and 95%CIs were used to assess the strength of association between IRS-2 gene rs1805097 polymorphism and CRC risk. Stratification analyses were carried out by ethnicity, SOC and cancer type. When a Q test indicated *P* < 0.1 or I^2^ > 50% indicated heterogeneity across studies, a random-effect model was used. Otherwise, the fixed-effects model was applied [22]. Allele model, dominant model, recessive model, homozygous model, and heterozygous model were used in this meta-analysis. We performed leave-one-out sensitivity analysis to evaluate the stability of the overall results. We assessed the departure from the HWE in the controls using Pearson's χ2 test. Potential publication bias was assessed by Begger's and Egger's linear regression test [23]; *P* < 0.05 was considered to indicate statistically significant. The power of this meta-analysis was calculated with a significant value of 0.05 [24].

## Abbreviations

CRC: colorectal cancer
IRS-2: insulin receptor substrate-2
IRS: insulin receptor substrates
IGF: insulin-like growth factor
IGFBP: insulin-like growth factor binding protein
IR: insulin resistance
SOC: source of control
CI: confidence interval
OR: odds ratio
NOS: Newcastle-Ottawa Scale
HWE: Hardy–Weinberg equilibrium
SNP: single nucleotide polymorphism
